# Personal

**Published:** 1903-10

**Authors:** 


### PERSONAL.

---

DR. WALTER B. DORSETT, of Saint Louis, announce the removal of his office, October 1, 1903, to the Linmar Building, (suite 26), southeast corner Washington and Vandeventer avenues. Hours:

1 to 4 p.m., 7 to 8 p.m., by appointment only. Both telephones at residence, 5070 Washington avenue. Office telephones: Linmar Building, Lin. 231, Kin. C 1363.

---

Dr. A. L. Benedict, of Buffalo, attended the meeting of the Canadian Medical Association at London, Ont., August 26, 1903, as delegate from the Medical Society of the State of New York. He also read a paper entitled, Multiple visceral lesion, at the annual meeting of the Erie County Medical Society, held at Erie, Pa., September 1, 1903.

---

Dr. William A. White, formerly assistant superintendent of the State Hospital for the Insane at Binghamton, N. Y., has been appointed by the Secretary of the Interior, Superintendent of the Government Hospital for the Insane at Washington, D. C., vice A. B. Richardson, deceased.

---

Dr. Eugene A. Smith, of Buffalo, who was ill for some weeks last spring, spent the summer in much needed rest, and resumed his professional work about September 1, 1903, much to the gratification of his colleagues and other numerous friends.

---

Dr. Montgomery A. Crockett, of Buffalo, who has been studying general surgery in Europe during the summer, has returned and resumed his professional work.

---

Dr. W. S. Bainbridge, of New York, delivered a series of lectures on medical history at Chautauqua during the last summer season.

---

Dr. and Mrs. J. V. Woodruff, of South Buffalo, are making a two months' tour of the Bermudas.

---

Dr. Irving M. Snow, of Buffalo, has returned after a few weeks of rest and recreation in Europe.

